# Identification of New Proteins and Potential Mitochondrial F_1_F_0_-ATPase Inhibitor Factor 1-Associated Mechanisms in *Arabidopsis thaliana* Using iTRAQ-Based Quantitative Proteomic Analysis

**DOI:** 10.3390/plants10112385

**Published:** 2021-11-05

**Authors:** Cuiting Chen, Yiqing Meng, Zhongyuan Hu, Jinghua Yang, Mingfang Zhang

**Affiliations:** 1 Laboratory of Germplasm Innovation and Molecular Breeding, Institute of Vegetable Science, Zhejiang University, Hangzhou 310058, China; chencuiting@zju.edu.cn (C.C.); mengyiqing@zju.edu.cn (Y.M.); mfzhang@zju.edu.cn (M.Z.); 2 Hainan Institute, Zhejiang University, Yazhou District, Sanya 572025, China

**Keywords:** iTRAQ, proteomic analysis, AtIF1, fertility, *Arabidopsis thaliana*, energy

## Abstract

The mitochondrial synthesis of ATP makes a vital contribution to the growth and development of biological organisms, in which the enzyme mitochondrial F_1_F_0_-ATP synthase plays a pivotal role, in that it can either synthesize or hydrolyze cellular ATP. The finding of our previous study revealed that mitochondrial F_1_F_0_-ATPase inhibitor factor 1 (IF1) in *Arabidopsis thaliana* has a conserved function as an endogenous inhibitor affecting cellular energy status and plays an important role in plant growth and reproduction, particularly in fertility. In this study, to gain an insight into IF1-related traits, we performed isobaric tags for relative and absolute quantitation labeling analysis. In total, 67 of 4778 identified proteins were identified as differentially expressed proteins (DEPs; 59 up-regulated and 8 down-regulated) between wild-type and *if1* mutant *Arabidopsis thaliana* seedlings. Gene ontology enrichment analysis revealed that these DEPs were the most significantly enriched in pathways such as “long-day photoperiodism, flowering,” “positive regulation of protein import into chloroplast stroma,” and “pollen sperm cell differentiation,” which are closely associated with reproductive development. Moreover, Kyoto Encyclopedia of Genes and Genomes enrichment analysis revealed that photosynthesis was the pathway most significantly enriched with DEPs. Collectively, our results revealed a global shift in protein abundance patterns corresponding to AtIF1 mutation, entailing changes in the abundance of multiple key proteins and metabolic processes, which will provide a valuable proteomic foundation for future studies.

## 1. Introduction

In mitochondria, F_1_F_0_-ATPase not only synthesizes the energy-bearing compound, ATP plays a physiological pH-dependent role in ATP hydrolysis [1,2]. In mammals and yeasts, the endogenous inhibitor mitochondrial F_1_F_0_-ATPase inhibitor factor 1 (IF1) has been shown to prevent the invalid hydrolysis of ATP by mitochondrial ATPase. Since its discovery, numerous studies have been conducted on IF1 in yeast and mammalian systems, particularly in human systems [3,4,5]. In yeast, IF1 inhibits the ATP hydrolysis activity of mitochondrial F_1_F_0_-ATPase when the impaired membrane potential is rectified, and ATP hydrolysis is not needed to ensure an adequate supply of ATP for cell physiological activity, which plays an important role in cell development [6,7]. In plants, IF1 was first isolated in potato (*Solanum tuberosum*; [8,9]), and subsequently, the sequence and subcellular localization of IF1 have been analyzed in rice (*Oryza sativa*; [10]). In a previous study, we characterized IF1 function in *Arabidopsis thaliana* and established that *Arabidopsis thaliana* IF1 (AtIF1) not only affects the energy status of cells but also plays important roles in growth and reproductive development, as mutation of this gene results in a decrease in dark-enhanced hypocotyl elongation and seed yield [11]. Although the findings of these studies have advanced our understanding of the physiological functions of IF1 in plants, we are far from clearly elucidating the mechanisms underlying these processes. In contrast to other eukaryotes such as yeasts and mammals, plants derive energy from both mitochondria and chloroplasts. Consequently, it is to be assumed that the molecular mechanisms and networks of IF1 in plant energy regulation are more complex than those in non-photosynthetic organisms.

Fertility encompasses a complex suite of traits regulated by multiple biological processes. In flowering plants, anther and pollen development are vital processes that directly determine the male fertility of the plants [12]. These developmental processes comprise several important steps, and a defect in any of these would interrupt pollen formation or disrupt male gametophytic function [13,14]. Given that pollen formation is a highly energy-consuming process [15], mitochondrial dysfunction in pollen grains was found to drastically affect pollen development [16]. Thus, it is not surprising that reduced fertility has previously been detected in *AtIF1* mutant lines [11]. Currently, little is known regarding the mechanisms underlying the activity of IF1 in plant reproductive development.

Over the past several years, the rapid development of proteomic technologies and the accumulation of large amounts of plant genome information have provided unprecedented opportunities for the global analysis of differences in the proteomes of diverse plant samples [17]. Different from nucleic acids, proteins are effector molecules, and, therefore, measuring these will contribute to a better understanding of the function and/or interactions of proteins in biological systems [18]. Isobaric tags for relative and absolute quantitation (iTRAQ) is a novel MS-based approach for the relative and absolute quantification of proteins using isobaric tags [19], which has advantages such as high-throughput, high sensitivity, good quantitation, and high accuracy [20]. To date, iTRAQ technology has been widely used in the study of various biological organisms, particularly model and major crop plants, such as *Arabidopsis* [21,22,23], tobacco [24], rice [25], maize [26], wheat [27], and cotton [28].

In this study, we applied iTRAQ technology for the first time to identify candidate proteins and potential metabolic processes that are directly associated with AtIF1 in *A. thaliana*. We identified a total of 4778 proteins, of which 67 (59 up-regulated and 8 down-regulated) were differentially expressed between wild-type and *if1* mutant samples. Bioinformation analysis indicated that the up- or down-regulated proteins in *if1* mutant samples were mainly involved in energy metabolism, particularly photosynthesis, and fertility-related processes, such as pollen development. These findings not only provided the potential functional proteins but also novel insights into the molecular mechanisms and networks underlying AtIF1-regulated fertility processes, which will constitute a proteomic foundation for future studies.

## 2. Materials and Methods

### 2.1. Plant Materials and Growth Conditions

As plant materials in the present study, we used wild-type *A. thaliana* (Columbia-0 ecotype) and *if1* mutant lines (SALK_000139) from the Arabidopsis Information Resource. The transgenic functional complementary lines (*p35S:AtIF1-if1*) were generated through expression of *AtIF1* in *if1* mutant line. More details of plant materials were shown in our previous study (Chen et al., 2020). Seeds were surface-sterilized for 4–12 h with Cl_2_ gas produced by the reaction between 5% (*v/v*) NaClO and concentrated HCl solution, after which they were seeded on 1/2 Murashige and Skoog medium (Sigma-Aldrich, St. Louis, MO, USA) with 0.8% agar and 1% sucrose. Following stratification treatment at 4 °C for 2–4 days under dark conditions, the plates were transferred to a plant growth chamber (Sanyo, Osaka, Japan) under long-day conditions, 16 h day (120 μmol m^−2^ s^−1^, 22 °C) and 8 h night (21 °C) at 55–60% relative humidity. Seedlings were grown for 4 weeks before analysis. Having collected whole plants, the samples were immediately frozen in liquid nitrogen and stored at −80 °C. Seven seedlings were harvested as one biological repeat. In total, 3 independent biological repeats were used for further analysis.

### 2.2. Extraction, Quantification, and Digestion of Cellular Proteins

Samples were ground into powder in liquid nitrogen and extracted with lysis buffer (7 M urea, 2 M thiourea, 4% 3-[(3-cholamidopropyl) dimethylammonio]-1-propanesulfonate (CHAPS), 40 mM Tris-HCl, pH 8.5) containing 1mM phenylmethylsulphonyl fluoride (PMSF), and 2 mM ethylene diamine tetra acetic acid (EDTA: final concentrations)). After 5 min, 10 mM dithiothreitol (DTT: final concentration) was added, and the suspension was sonicated at 200 Watt (W) power for 15 min, then centrifuged at 4 °C for 15 min at 30,000× *g*. The resulting supernatant was mixed well with a 5× volume of chilled acetone containing 10% (*v/v*) trichloroacetic acid and incubated at −20 °C overnight. Following further centrifugation, the supernatant was discarded and the remaining precipitate was washed three time with chilled acetone. After air drying, the pellet was dissolved in lysis buffer (7 M urea, 2 M thiourea, 4% NP40, 20 mM Tris-HCl, pH 8.0–8.5). The suspension was sonicated at 200 W for 15 min and centrifuged at 4 °C for 15 min at 30,000× *g* with the resulting supernatant being transferred to a fresh tube. To reduce disulfide bonds in the supernatant proteins, 10 mM DTT (final concentration) was added, followed by incubation at 56 °C for 1 h. Subsequently, 55 mM iodoacetamide (IAM; final concentration) was added and incubated in the dark for 1 h. Thereafter, the supernatant was mixed well with a 5× volume of chilled acetone at −20 °C for 2 h to precipitate proteins. Following centrifugation, the supernatant was discarded, and the pellet was air-dried for 5 min, dissolved in 500 μL 0.5 M TEAB (Applied Biosystems, Milan, Italy), and sonicated at 200 W for 15 min. After further centrifugation at 4 °C for 15 min at 30,000× *g*, the resulting supernatant was transferred to a fresh tube and stored at −80 °C for further analysis. Total protein concentrations were determined using the Bradford assay with bovine serum albumin (BSA) used as a standard [29], and samples were subsequently digested at 37 °C for 16 h using Trypsin Gold (Promega, Madison, WI, USA) at a protein–trypsin ratio of 30:1.

### 2.3. iTRAQ Labeling and Strong Cation Exchange Fractionation

Samples used for iTRAQ analysis were labeled in strict accordance with the instructions provided with the 8-plex iTRAQ reagent (Applied Biosystems, Foster City, CA, USA). Strong cation exchange (SCX) chromatography was performed using an LC-20AB HPLC Pump system (Shimadzu, Kyoto, Japan), as described by Wu et al. [24].

### 2.4. Liquid Chromatography–Tandem Mass Spectrometry Analysis

Each of the SCX fractions was resuspended in buffer A (5% acetonitrile (]an), 0.1% formic acid (FA)) and centrifuged at 20,000× *g* for 10 min, with the final concentration of peptides being, on average, approximately 0.5 μg/μL. Using an autosampler, 10 μL supernatant was loaded onto a 2 cm C18 trap column of an LC-20AD nanoHPLC system (Shimadzu, Kyoto, Japan). The peptides were then eluted onto a 10 cm analytical C18 column (inner diameter 75 μm) packed in-house. The samples were loaded at 8 μL/min for 4 min, after which a 35 min gradient was run at 300 nL/min, starting from 2% to 35% buffer B (95% ACN, 0.1% FA), followed by a 5 min linear gradient to 60%, a 2 min linear gradient to 80%, maintenance at 80% buffer B for 4 min, and, finally, a return to 5% for 1 min.

Data acquisition was performed using a TripleTOF 5600 System (AB SCIEX, Concord, ON) fitted with a Nanospray III source (AB SCIEX, Concord, ON) and a pulled quartz tip as the emitter (New Objectives, Woburn, MA). Data were acquired using an ion spray voltage of 2.5 kV, curtain gas pressure of 30 psi, nebulizer gas pressure of 15 psi, and an interface heater temperature of 150 °C. The MS was with an RP of greater than or equal to 30,000 FWHM for TOF MS scans. For IDA, survey scans were acquired in 250 ms, and as many as 30 product ion scans were collected if they exceeded a threshold of 120 counts per second (counts/s) and with a 2+ to 5+ charge state. The total cycle time was fixed to 3.3 s. Q2 transmission window was 100 Da for 100%. Four-time bins were summed for each scan at a pulser frequency value of 11 kHz by monitoring of the 40 GHz multichannel TDC detector with a four-anode channel detection ion. A sweeping collision energy setting of 35 ± 5 eV coupled with iTRAQ-adjusted rolling collision energy was applied to all precursor ions for collision-induced dissociation. Dynamic exclusion was set at 1/2 of the peak width (15 s), and then the precursor was refreshed off the exclusion list.

### 2.5. Data and Bioinformatics Analysis

In the present study, we identified proteins using the modular approach to software construction operation and test (MASCOT) search engine (Matrix Science, London, UK; version 2.3.02), which contains 129,464 sequences from the Arabidopsis UniProt database (http://www.uniprot.org/uniprot/?Query=taxonomy:3701 (accessed on 5 January 2018)). To reduce the identification of pseudopeptides, only peptides with a significant score (≥20) higher than the 99% confidence interval of “identity” were counted as identified proteins through the MASCOT probability analysis. For protein quantification, proteins should contain at least two unique peptides. For the MASCOT probability analysis, the quantitative protein ratio was weighted and normalized to the median ratio. We used a ratio of *p*-value < 0.05 and a multiple change of *p*-value > 1.2 to indicate significantly differentially expressed proteins (DEPs). The Blast2GO program was used to annotate DEPs in the non-redundant protein database (NR; NCBI), Kyoto Genome and Genome Encyclopedia (KEGG), and COG (the direct homologous group of proteins) databases.

### 2.6. RNA Extraction and Real-Time Quantitative PCR

Total RNA was extracted using an RNAprep Pure Plant Kit (TIANGEN, DP432) and was subsequently reverse transcribed into cDNA using a ReverTra Ace qPCR RT Kit (TOYOBO, Osaka, Japan). Real-Time quantitative PCR (RT-qPCR) reactions were carried out using an ABI StepOne Real-Time PCR system (Applied Biosystems, Foster City, CA, USA), with fold changes of the selected genes being normalized to that of the *actin 2* reference gene. The relative expression levels of target genes were determined using the 2^−ΔΔCT^ method (Livak and Schmittgen, 2001), and the primers used for amplification are listed in Supplementary Table S1. All PCR experiments were performed using three independent biological replicates, and significant differences (*p* < 0.05) in the data were analyzed using Student’s *t*-test.

## 3. Results

### 3.1. Analysis of A. thaliana Protein Profiles Using iTRAQ

**Figure 1:**
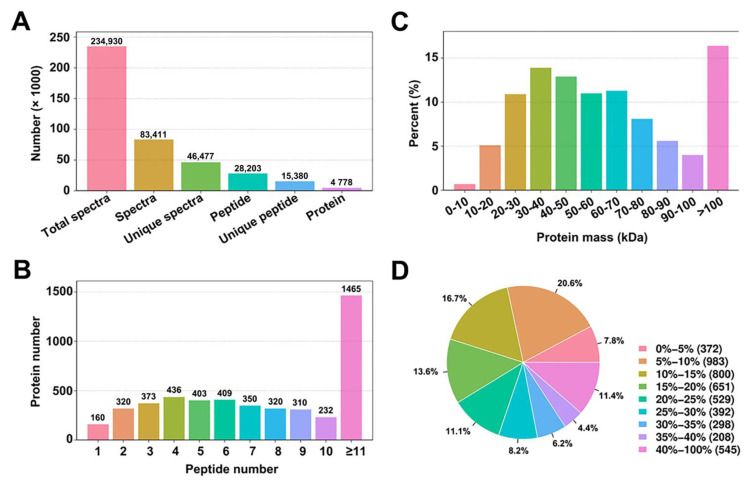
Primary data analysis and protein identification. (**A**). Basic information statistics of peptide and proteins. (**B**). Peptide number distribution. (**C**). Protein mass distribution. (**D**). Protein sequence coverage distribution.

To examine total protein changes in the protein profiles and to gain a global view of the cellular processes occurring in response to *AtIF1* mutation, we performed comparative proteomics analysis using a multiplex iTRAQ technique in *A. thaliana* wild-type (WT) and T-DNA knockout *if1* mutant 4-week-old seedlings. At this stage, both lines are in the transitional period and show similar vegetative organs (Supplementary Figure S1). The peptide and quality information for the identified proteins are shown in Figure 1. We obtained a total of 234,930 spectra. To identify the proteins from the mass spectrometry data, we employed MASCOT, a powerful database retrieval software, to generate a total of 83,411 spectra matched to in silico peptide spectra, which revealed 46,477 unique spectra, 28,203 peptides, 15,380 unique peptides, and 4778 proteins from 3 independent biological repeats (Figure 1A). Additionally, among the proteins identified, 160 had one unique peptide, 320 had two, 373 had three, and 1465 had more than 11, with the remainder having 4–10 (Figure 1B). The peptide information validated that many unique peptides are shared with different proteins (the identified peptides were compared with protein databases). Upon examination of the statistics of all identified proteins, based on the relative molecular mass (Figure 1C), it was revealed that the relative molecular mass of the identified proteins was primarily concentrated in the 20–80 kDa range, although more than 15% of the proteins had molecular masses greater than 100 kDa. Furthermore, we identified a total of 3335 proteins with 0–25% sequence coverage, whereas only 4.35% of the identified proteins had a sequence coverage of 35–40% (Figure 1D).

### 3.2. Identification of Proteins Differentially Expressed between A. thaliana Wild-Type and the if1 Mutant Lines

In total, we identified 4778 proteins involved in a wide range of metabolic and signaling pathways, including post-translational modification, protein turnover, chaperones, general function prediction only, translation, ribosomal structure and biogenesis, carbohydrate transport and metabolism, energy production and conversion, amino acid transport and metabolism, and so on (Supplementary Figure S2). Based on the level of protein abundance, the proteins were screened using the criteria of a fold-change value >1.2 or <0.8333 and a *p*-value < 0.05 as the DEPs. In total, 67 DEPs in response to *AtIF1* mutation were obtained, of which 59 DEPs were up-regulated and 8 DEPs were down-regulated in the *A. thaliana if1* mutant compared with that in the WT (Table 1). These results indicated that the *AtIF1* mutation had a marked effect on the *A. thaliana* proteome, resulting in the differential accumulation of these proteins.

### 3.3. Functional Annotation of Identified Proteins with Differential Accumulation in A. thaliana Wild-Type and if1 Mutant Plants

**Figure 2:**
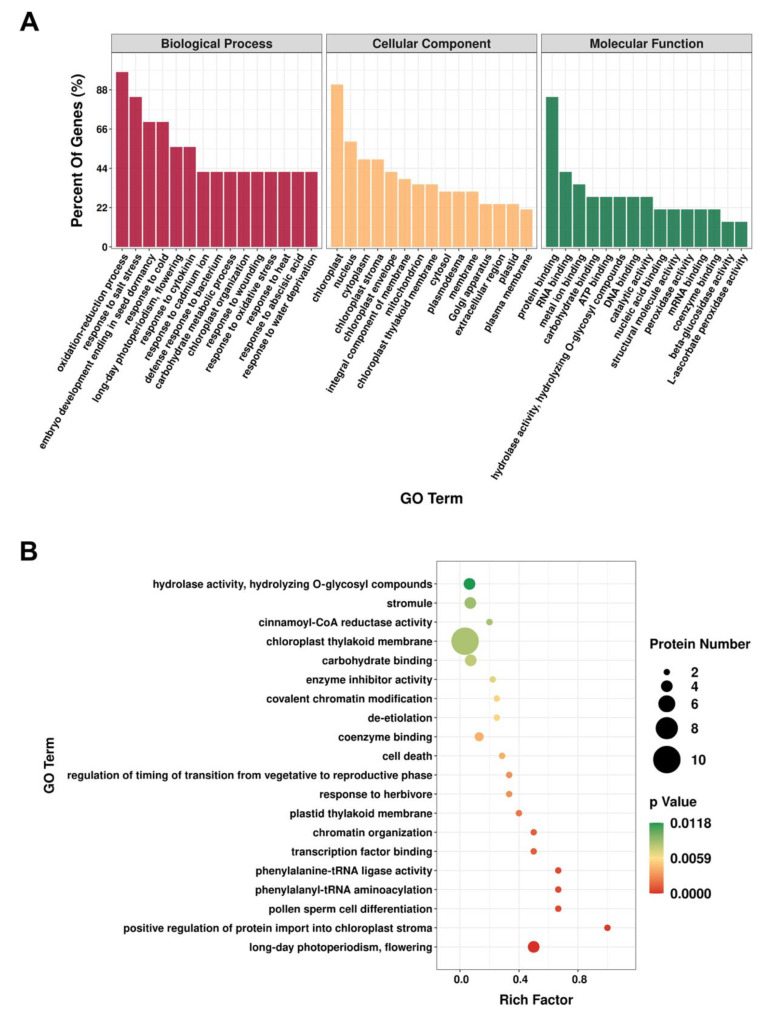
Gene Ontology (GO) analysis of the differentially expressed proteins (DEPs) in *Arabidopsis thaliana*. (**A**). GO categories for DEPs in the proteome. (**B**). GO enrichment analysis of DEPs.

To gain an overall understanding of the proteomic changes in the absence of AtIF1, we conducted GO annotations of the DEPs screened between *A. thaliana* WT and *if1* mutant plants (*p*-value ≤ 0.05) (Figure 2). DEPs were categorized by biological process (BP), cellular component (CC), and molecular function (MF). The results showed that 30, 12, and 23 GO terms were enriched for biological processes, cellular components, and molecular functions, respectively (Supplementary Table S2). With respect to biological processes, the three functional categories with the highest number of DEPs were “oxidation-reduction process”, “response to salt stress”, and “embryo development ending in seed dormancy”. Among the cellular component functions, the identified DEPs were mainly related to “chloroplast”, “nucleus”, and “cytoplasm”. For molecular functions, most DEPs were found to be associated with “protein binding”, “RNA binding”, and “metal ion binding” (Figure 2A). We subsequently performed GO enrichment analysis based on proteins with *p*-values < 0.05, which revealed that the categories “long-day photoperiodism, flowering”, “positive regulation of protein import into chloroplast stroma”, “pollen sperm cell differentiation”, “phenylalanyl-tRNA aminoacylation”, and “chromatin organization” were the most significantly enriched (Figure 2B).

### 3.4. KEGG Pathway Analysis of the DEPs

**Figure 3:**
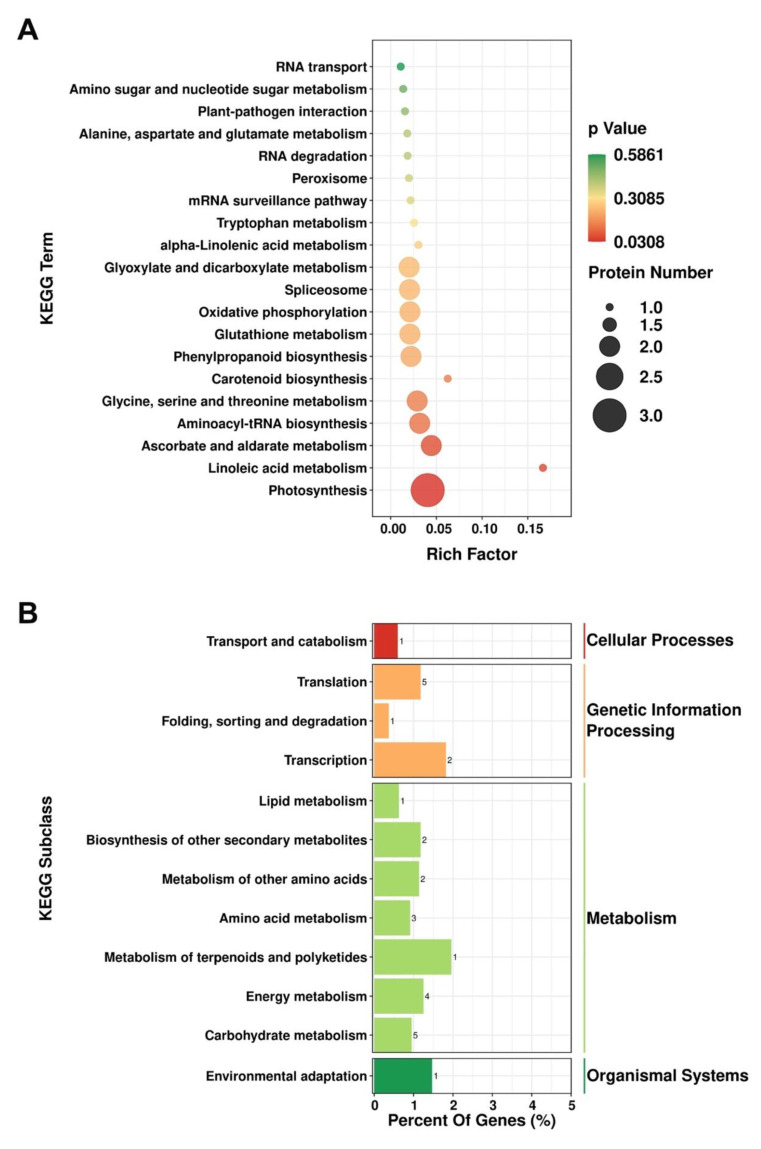
Kyoto Encyclopedia of Genes and Genomes (KEGG) pathway enrichment of differentially expressed proteins (DEPs) in *Arabidopsis thaliana*. (**A**). Bubble chart of the top 20 pathways in KEGG pathway analysis of DEPs. (**B**). KEGG enrichment analysis of DEPs.

To evaluate the role of the DEPs detected based on iTRAQ analysis in the different pathways, we mapped these proteins to KEGG pathways for enrichment analysis. We accordingly assigned the DEPs to 28 pathways, among which, “Linoleic acid metabolism”, “Photosynthesis”, and “Systemic lupus erythematosus” constituted the three highest enriched pathways with *p*-values < 0.05 (Figure 3A). The identified DEPs were found to be primarily involved in metabolic processes, including “carbohydrate metabolism” (five proteins), “energy metabolism” (four proteins), “metabolism of terpenoids and polyketides” (one protein), “amino acid metabolism” (three proteins), “metabolism of other amino acids” (two proteins), “biosynthesis of other secondary metabolites” (two proteins), and “lipid metabolism” (one protein) (Figure 3B).

### 3.5. Analysis of the Transcriptional Expression of the DEPs

**Figure 4:**
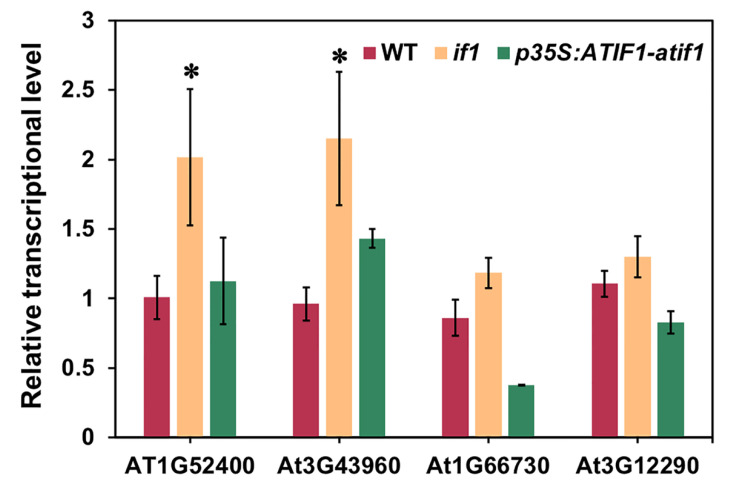
Real-Time qPCR analysis of the DEPs from iTRAQ analysis in *A. thaliana* WT, *if1*, and *p35S:AtIF1-if1* complementary lines. Data represent the means ± SEs of three independent experiments. Asterisks indicate the significance level according to Student’s *t*-test: * *p* < 0.05.

To verify the changes in protein accumulation determined using iTRAQ analysis, we performed Real-Time qPCR analysis for selected genes in WT, *if1*, and *p35S:AtIF1-if1 A. thaliana* plants. Since AtIF1 was identified in our pervious study to affect cellular energy state, we selected some genes related to energy metabolism for qPCR verification. As shown in Figure 4, the transcripts of three genes (*At1G52400/BGLU18*, *AT3G43960*, and *AT1G66730/ATLIG6*) showed elevated accumulation in the *if1* mutant, which is consistent with the trends of changes in protein patterns based on iTRAQ analysis. Only a single gene (*AT3G12290/MTHFD1*) showed minor differences in transcriptional abundance between WT and *if1* mutant plants, which we assume to be attributable to post-transcriptional regulation, as documented in other proteomic studies [30]. The transcription levels of these genes in *p35S:AtIF1-if1* plants also verified the iTRAQ result again.

## 4. Discussion

### 4.1. Proteomic Changes between A. thaliana Wild-Type and if1 Mutant Seedlings

Proteomic analysis is a powerful tool that provides high-throughput information relating to the protein-level mechanisms underlying different biological processes, such as autophagy, anther and pollen development, and chloroplast development [12,22,30,31,32]. In this study, we conducted a comparative whole-plant proteomics analysis of *A. thaliana* WT and *if1* seedlings to identify potential AtIF1-related proteins and mechanisms. The relative quantitative changes in protein abundance determined using an iTRAQ-based method and 67 identified DEPs may be regulated to a greater or lesser extent by AtIF1 (Table 1). The limited number of DEPs detected is likely due to the healthy growth status of the examined *A. thaliana* seedlings under normal conditions. Nevertheless, 86.57% of the DEPs in the *if1* mutant seedlings were found to be significantly up-regulated, which is indicative of the fact that a range of metabolic activities are negatively regulated by AtIF1.

### 4.2. Identification of AtIF1-Regulated Proteins That Involved in Energy Metabolism

In our previous study, we found that AtIF1 influences the energy status of *A. thaliana* cells [11]. Not surprisingly, in the present study, a number of DEPs were identified by iTRAQ-based proteomics analyses, which are closely involved in energy metabolism, such as pathways of photosynthesis and oxidative phosphorylation (Figure 3). Photosystem II is a large membrane protein complex that catalyzes the light-driven electron transfer from water to plastoquinone, thereby generating electrons for the entire photosynthetic electron transport chain [33]. Among the identified DEPs, a chloroplast-encoded photosystem II reaction center protein D (PsbD/ATCG00270; [34]) and a distinct hydrophilic photosystem II assembly protein (Psb28/AT4G28660; [35]) were found to be significantly up-regulated in *if1* mutant seedlings. Moreover, KEGG enrichment analysis similarly revealed that photosynthesis, with the highest number of associated DEPs, was the most significantly enriched pathway (Figure 3A). These findings indicate that mutations in *AtIF1* may affect the cellular energy status of *A. thaliana* via its involvement in both photosynthesis and respiration pathways. Indeed, the findings of previous studies have indicated that IF1 inhibits the invalid hydrolysis of ATP by the mitochondrial F_1_F_0_-ATPase in other eukaryotes, such as yeasts and mammals, to regulate cellular energy status [11]. To the best of our knowledge, the present study is the first to demonstrate a tight association between photosynthesis and IF1-mediated energy processes in *A. thaliana*, which will advance our current understanding of the molecular mechanisms and networks of plant IF1s in energy regulation and warrants further study.

### 4.3. Identification of AtIF1-Regulated Proteins Involved in Reproductive Development

We have previously established that the *if1* mutant of *A. thaliana* is characterized by reduced fertility [11]. However, no significant difference could be observed in the pollen production and its viability between two lines (Figure S3). Since silique length is also decreased in the mutant, the reduced seed yield in the *if1* mutant [11] may not be, at least not solely, due to the decreased pollen number or activity. We supposed that the altered protein pattern, resulting from *IF1* mutation, might already start to affect the incoming reproductive development before the formation of the floral organ. Consistently, our comparative iTRAQ protein profiling and the subsequent GO functional analyses in the present study revealed that the GO terms such as “long-day photoperiodism, flowering”, “pollen sperm cell differentiation”, and “regulation of timing of transition from vegetative to reproductive phase” were significantly enriched (Figure 2, Supplementary Table S2). Among these proteins, BRI1-EMS-SUPPRESSOR 1 (BES1), a key transcription factor for brassinosteroid signaling, has been found to be involved in plant reproduction and seed development [36,37]. Using chromatin immunoprecipitation assay(ChIP)analysis, Ye et al. [38] demonstrated that BES1 can bind directly to the promoter regions of several key genes required for anther and pollen development in *A. thaliana*, including *SPOROCYTELESS/NOZZLE* (*SPL/NZZ*), *TAPETAL DEVELOPMENT AND FUNCTION 1* (*TDF1*), *MALE STERILITY 1* (*MS1*), and *MS2*. Thus, the down-regulation of the BES1 proteins in *if1* mutant *A. thaliana* seedlings is likely to contribute to the observed reduction in fertility. It is known that phenylpropanoid is very important for cell wall formation in pollen grains and is crucial for normal anther development [39]. Additionally, the biosynthesis of sporopollenin, the main component of the pollen exine, is also closely related to phenylpropanoid metabolism [40,41]. In this study, we found the phenylpropanoid biosynthesis pathway to be significantly enriched with the DEPs in WT and *if1* seedlings (Figure 3A). The overaccumulation of several key proteins of phenylpropanoid biosynthesis in *if1* mutants would appear to be indicative of either an abnormality in pollen cell wall formation, which may lead to a reduced fertility, or a feedback regulation on phenylpropanoid synthesis due to defects in pollen development. Overall, these findings provide new insights into the molecular mechanisms underlying the AtIF1-regulated fertility.

## 5. Conclusions

Collectively, the findings of this study provided a global profiling of proteins regulated by AtIF1. We established that numerous key DEPs were significantly enriched in the pathways associated with energy metabolism and reproductive development, which we presume contributes to changes in cellular energy status and a significant reduction in the seed yield of the *A. thaliana if1* mutant [11]. Our findings provide novel insights into the molecular mechanisms underlying AtIF1-regulated energy and fertility processes, as well as laying a valuable proteomic foundation for future studies on IFs in plants.

## Figures and Tables

**Table 1:** Detailed information of the DEPs between *A. thaliana* WT and *if1* mutant lines.

Accession	Protein Description	Gene Name	Fold Change (*if1*/WT)
**Up-regulated DEPs**			
A0A178W6T5_ARATH	HIP1.3	At1g09240	1.21
A0A0U5CLC7_9BRAS	Photosystem II D2 protein	psbD	1.29
A0A178V4M1_ARATH	RBP31	At4g28660	1.29
JAL28_ARATH	Nitrile-specifier protein 1	NSP1	1.21
BGL37_ARATH	Myrosinase 2	TGG2	1.34
A0A178W5I1_ARATH	L-ascorbate peroxidase	At1g07590	1.24
D7KHW7_ARALL	L-ascorbate peroxidase	ARALYDRAFT_470824	1.24
A0A178V395_ARATH	PAP_fibrillin domain-containing protein	At4g25850	1.22
D7L023_ARALL	Alanine--glyoxylate aminotransferase	ARALYDRAFT_480305	1.23
D7M155_ARALL	Plastid lipid-associated protein 1, chloroplast	ARALYDRAFT_911682	1.22
LOX2_ARATH	Lipoxygenase 2, chloroplastic	LOX2	1.25
JAL30_ARATH	PYK10-binding protein 1	PBP1	1.2
A0A178VSK5_ARATH	Uncharacterized protein	At2g41150	1.25
A0A178VBA5_ARATH	Glutaredoxin-dependent peroxiredoxin	At3g05750	1.21
RL61_ARATH	60S ribosomal protein L6-1	RPL6A	1.26
A0A178V6D3_ARATH	Phenylalanyl-tRNA synthetase	At3g52700	1.21
A0A178WET2_ARATH	-	-	1.22
A0A178VVV5_ARATH	THF1	At2g16340	1.21
D7MSA7_ARALL	Uncharacterized protein	ARALYDRAFT_918349	1.47
A0A178V4T2_ARATH	HMA domain-containing protein	At4g33520	2.02
A0A178UEX5_ARATH	CDF1	At5g22550	1.25
D7M1M2_ARALL	Uncharacterized protein	GN=ARALYDRAFT_489157	1.25
Q1H5F7_ARATH	Histone H2B	-	1.24
A0A178WJ12_ARATH	TLL1	At1g40860	1.26
A0A178UTL8_ARATH	XYL4	At5g64200	1.21
A0A178VST0_ARATH	JAL22	At2g36220	1.26
A0A178UDT2_ARATH	SAG15	At5g49860	1.28
BGL18_ARATH	Beta-D-glucopyranosyl abscisate beta-glucosidase	BGLU18	1.24
FAX2_ARATH	Protein FATTY ACID EXPORT 2, chloroplastic	FAX2	1.21
A0A178WJL7_ARATH	Phenylalanyl-tRNA synthetase beta subunit	At1g66730	1.2
A0A178VZH7_ARATH	Histone H2B	At2g24830	1.27
A0A178U9K8_ARATH	OPT4	At5g64020	1.26
A0A178UG56_ARATH	Uncharacterized protein	At5g46060	1.31
A0A178W4H7_ARATH	Uncharacterized protein	At1g74710	1.22
A0A178UH43_ARATH	-	-	1.21
A0A178WIZ0_ARATH	Lipase_GDSL domain-containing protein	At1g48430	1.21
A0A178WEL1_ARATH	ATP-dependent Clp protease proteolytic subunit	At1g12090	1.25
A0A178WD65_ARATH	ATARP4	At1g19350	1.23
D7KGE2_ARALL	Uncharacterized protein	ARALYDRAFT_472063	1.23
A0A178V8P6_ARATH	Methionine aminopeptidase 2	At3g54390	1.2
D7KBN6_ARALL	Uncharacterized protein	ARALYDRAFT_472926	1.21
A0A178W3R8_ARATH	Uncharacterized protein	At1g27300	1.21
A0A178W7M1_ARATH	Uncharacterized protein	At1g29860	1.24
A0A178UX37_ARATH	SGT1B	At4g12690	1.25
D7LBM5_ARALL	tRNA pseudouridine synthase family protein	ARALYDRAFT_481943	1.83
A0A178VSZ1_ARATH	JAL23	At2g36240	1.27
A0A178UZA3_ARATH	SSR16	At4g39620	1.23
A0A178UP34_ARATH	Epimerase domain-containing protein	At5g58490	1.25
A0A178W827_ARATH	KTI1	At1g67490	1.39
A0A178WIQ3_ARATH	RZ-1b	At1g53730	1.23
Q6NPL0_ARATH	At4g09040	At4g09040	1.22
A0A178VFH3_ARATH	Uncharacterized protein	At3g12290	1.24
A0A178V9V7_ARATH	FTM4	At3g12170	1.35
A0A178WN71_ARATH	Plastocyanin	At1g70550	1.25
D7MQE2_ARALL	Cinnamoyl-CoA reductase family	ARALYDRAFT_495968	1.29
A0A178WC08_ARATH	SRZ21	AXX17_At1g25030	1.33
Y4554_ARATH	Uncharacterized protein At4g15545	At4g15545	1.24
D7KZQ1_ARALL	Leucine-rich repeat protein FLR1	ARALYDRAFT_897426	1.26
A0A178V6Y8_ARATH	BSD domain-containing protein	AXX17_At3g43960	1.22
**Down-regulated DEPs**			
A0A178WA55_ARATH	Uncharacterized protein	At1g50990	0.8
A0A068CL28_ARALY	At4g21450p-like protein	-	0.8
A0A178V1L3_ARATH	PDE332	At4g10860	0.82
A0A178V0A8_ARATH	Nodulin-like domain-containing protein	At4g39930	0.68
A0A178UJR2_ARATH	DegP10	At5g33920	0.79
A0A178VP21_ARATH	ANK_REP_REGION domain-containing protein	At3g03550	0.25
D7MFW8_ARALL	Xyloglucan endotransglucosylase/hydrolase	ARALYDRAFT_354208	0.73
A0A178WAG2_ARATH	Uncharacterized protein	AXX17_At1g08440	0.77

## Data Availability

All data represented in this study are available in this article and Supplementary Materials.

## Supplementary Materials

The following are available online at https://www.mdpi.com/article/10.3390/plants10112385/s1, Figure S1: Phenotype of WT, *if1* and *p35S:AtIF1-if1* complementary lines under normal growth condition. Figure S2: The GO classification, KOG classification, and KEGG pathways analysis of identified proteins of WT and *if1* mutant *A. thaliana* lines. Figure S3: Pollen analysis on WT and *if1* mutant lines. Table S1: Primers used in this study. Table S2: Detail information of the GO annotation by agriGO analysis.

## Abbreviations

IF1, inhibitor factor 1; *A. thaliana, Arabidopsis thaliana*; iTRAQ, isobaric tags for relative and absolute quantitation; DEPs, differentially expressed proteins; GO, Gene ontology; KEGG, Kyoto Encyclopedia of Genes and Genomes; WT, wild-type; MASCOT, Modular Approach to Software Construction Operation and Test; RT-qPCR, Real-Time quantitative PCR; PsbD, Photosystem II reaction center protein D; BES1, BRI1-EMS-SUPPRESSOR 1.
