# Supplementary material for: Identification of New Proteins and Potential Mitochondrial F1F0-ATPase Inhibitor Factor 1-Associated Mechanisms in Arabidopsis thaliana Using iTRAQ-Based Quantitative Proteomic Analysis
Source: Plants (Basel). 2021 Nov 5;10(11):2385. doi: 10.3390/plants10112385 (PMC8619367; doi:10.3390/plants10112385)
Supplement: Supplementary file 1 [file plants-10-02385-s001.zip › plants-1395101-supplementary.pdf]

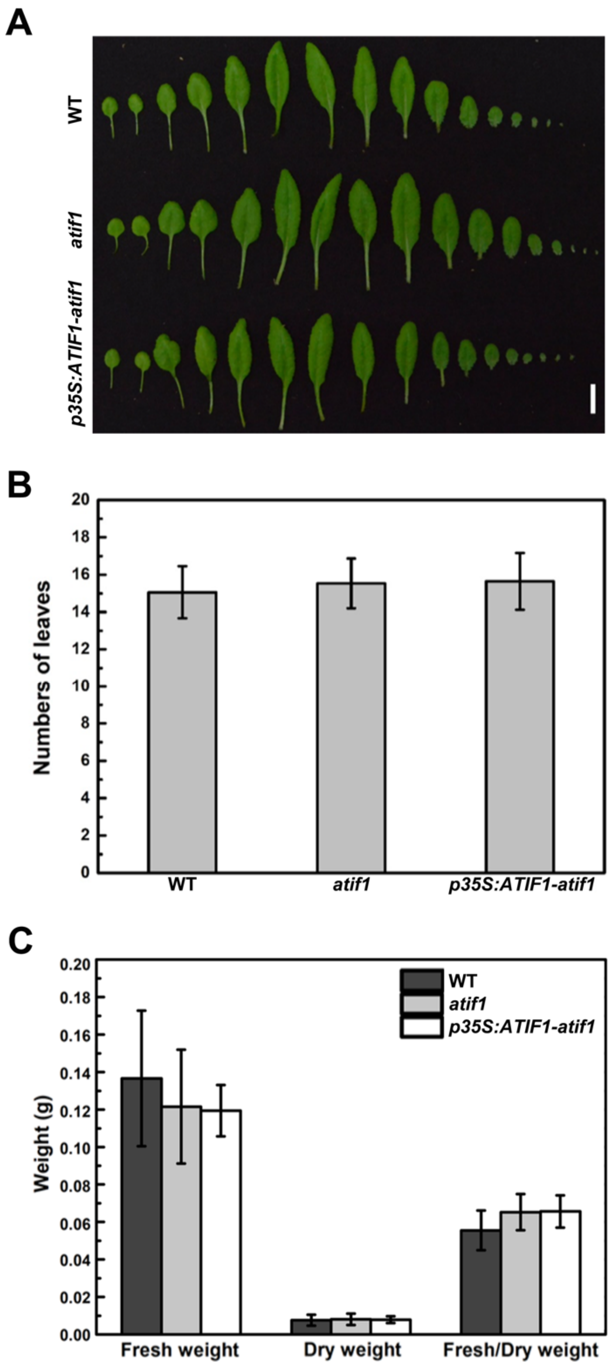

Figure S1. Phenotype of WT, *if1* and *p35S:AtIF1-if1* complementary lines under normal growth condition. (A) Leaves of 4-week-old seedlings. Bars = 1 mm. (B) Number of leaves of 4-week-old seedlings. (C) Weight of 4-week-old seedlings.

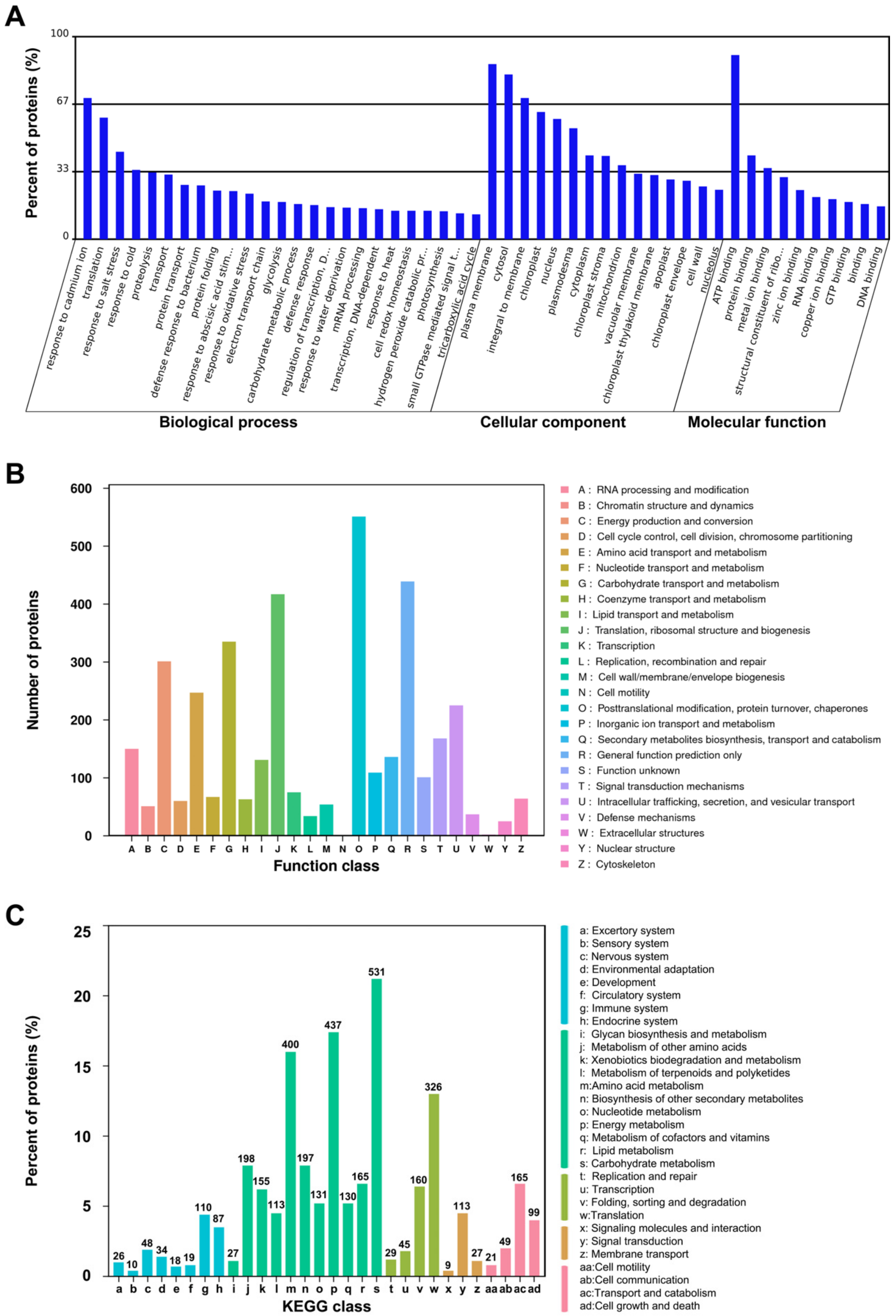

Figure S2. The GO classification (A), KOG classification (B), and KEGG pathways analysis (C) of identified proteins of WT and *if1* mutant *A. thaliana* lines.

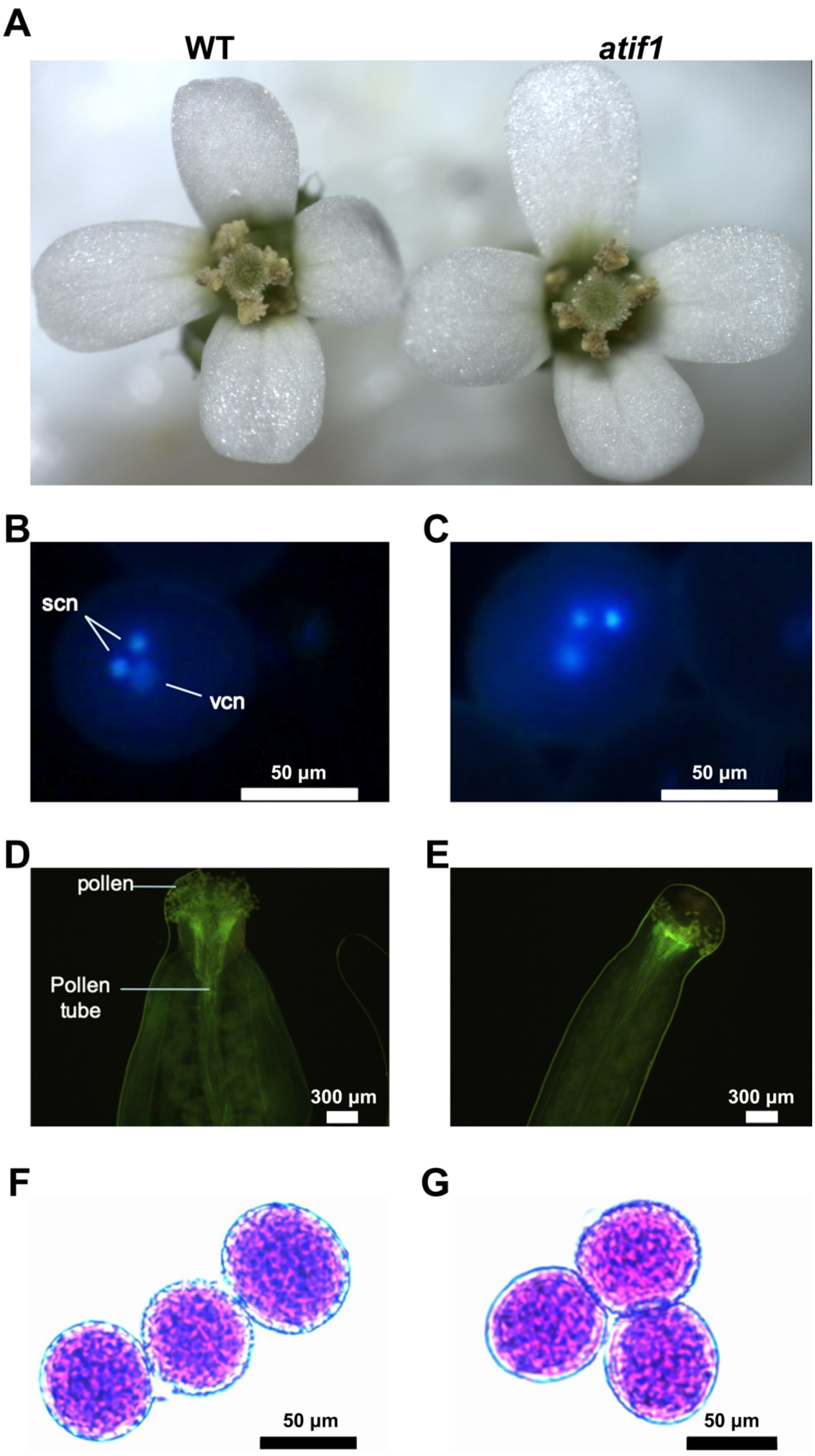

Figure S3. Pollen analysis of WT and *if1* mutant lines. (A) Flowers. (B) and (C) 4′,6-Diamidino-2-phenylindole-stained pollen. One vegetative cell nucleus (vcn) and two sperm cell nuclei (scn) are observed in WT (B) and *if1* mutant lines (C) pollen. Bars = 50 µm. (D) and (E) Aniline blue-stained pistils of WT (D) and *if1* mutant (E) lines flowers. Bars = 300 µm. (F) and (G) Alexander staining for pollen viability of WT (F) and *if1* mutant (G) lines. Bars = 50 µm;
