# Supplementary material for: Identification of New Proteins and Potential Mitochondrial F1F0-ATPase Inhibitor Factor 1-Associated Mechanisms in Arabidopsis thaliana Using iTRAQ-Based Quantitative Proteomic Analysis
Source: Plants (Basel). 2021 Nov 5;10(11):2385. doi: 10.3390/plants10112385 (PMC8619367; doi:10.3390/plants10112385)
Supplement: Supplementary file 1 [file plants-10-02385-s001.zip › Table S1 Primers used in this study.pdf]

Table S1. Primers used in this study.

Primer Name	Forward Primer (5'-3')	Reverse Primer (5'-3')
At1g66730-qPCR	TCTCTGGACGTGGTTGTTCCTT	GCGCGCTGGCCATCATATTT
AT1G52400-qPCR	TGCAAACAGTGTGCTGGAGGT	TGACCGACACGATCCTTCATCG
At3g12290-qPCR	GGGCAACGAGCAGTTGTTGT	TGCCCTTAATCATGTGGGCTTGT
At3g43960-qPCR	AGGAGAGTGTGGAAGTTGTTGGG	CCCATACTGCTCCACCACCAG
Actin2-qPCR	TATCGCTGACCGTATGAGCAAAG	TGGACCTGCCTCATCATACTCG
